# Open reduction and internal fixation of irreducible displaced femoral neck fracture with femoral Neck System: a preliminary study

**DOI:** 10.1186/s12891-023-06839-3

**Published:** 2023-10-19

**Authors:** Chengzhi Liang, Yuan Cao, Zhihao Lin, Guoming Liu, Chengdong Zhang, Yanling Hu

**Affiliations:** 1 Department of Orthopedics, Affiliated Hospital of Qingdao University, Qingdao University, Qingdao, Shandong 266003 People’s Republic of China; 2 Department of Orthopaedic Surgery, The Second Affiliated Hospital of Nanchang University, Nanchang, Jiangxi 330000 People’s Republic of China; 3 Department of Orthopedics, People’s Hospital of Rizhao, Rizhao, Shandong 276800 People’s Republic of China

**Keywords:** Displaced femoral neck fracture, Femoral neck system (FNS), Direct anterior approach (DAA), Open reduction

## Abstract

**Background:**

Most displaced femoral neck fractures can achieve satisfactory anatomical reduction by closed reduction, but there are still some that cannot reset satisfactorily after closed reduction, and open reduction are required. Such fractures that cannot be repositioned successfully by closed reduction are called irreducible displaced femoral neck fractures in this study. The objective of our study was to evaluate the efficacy of direct anterior incision with the Femoral Neck System in the treatment of irreducible displaced femoral fractures.

**Methods:**

A total of 16 young and middle-aged patients with irreducible displaced femoral neck fractures involving Garden type III and IV were treated using Femoral Neck System fixation by open reduction through Direct Anterior Approach between January 2020 to September 2021. Functional outcomes and postoperative complications were assessed during follow-up. Clinical outcomes were evaluated by the Hip Harris score. The postoperative reduction was evaluated by the Garden Index. Observe postoperative complications.

**Results:**

All patients were followed up with a mean follow-up time of 21.1(12–30) months, and according to radiological results, all patients achieved fracture healing, with a mean healing time of 4.25 months. All 16 patients received grade Garden I and II reductions, and there was no significant difference in the anteroposterior Garden reduction index between the first day after surgery (166.13 ± 5.61) and the 12th month after surgery(164.94 ± 4.49) (P>0.05) and no significant difference in lateral Garden index between the first day after surgery(171.06 ± 4.46) and the 12th month after surgery(169.38 ± 3.98) (P<0.05). According to the Hip Harris score scale, 13 patients received excellent and 3 patients received good. The postoperative Hip Harris Score(17.19 ± 4.8) was significantly higher than the preoperative score(92.19 ± 3.4), and the difference was statistically significant (P < 0.05). No or mild femoral neck shortness occurred in 12 (75%) patients, moderate shortening occurred in 3 (18.75%) patients, and severe shortening occurred in 1 (6.25%) patient. None of the patients experienced femoral head necrosis, fracture nonunion, or incision infection. One patient developed deep venous thrombosis of the lower extremity.

**Conclusions:**

The Direct Anterior Approach combined with Femoral Neck System is an excellent treatment for irreducible displaced femoral neck fracture and achieved good functional outcomes and anatomical reduction with low complications.

## Introduction

Femoral neck fractures (FNFs) in patients under the age of 50 years account for less than 5% of all hip fractures and are typically the result of a high-energy event [1, 2]. FNFs in young patients, particularly displaced fractures, are challenging to treat. Internal fixation remains the consensus, and the quality of the reduction is more important than the time to surgery [3, 4]. Closed repositioning is the most commonly used method in clinical practice. When closed manipulation is inadequate for anatomic reduction, such as when soft tissue is embedded in the fracture end, an open surgical approach is required. Our goal is to achieve anatomic reduction through open or closed reduction.

The main areas of interest in the management of open reduction are the choice of the surgical approach and the implant. The direct anterior approach (DAA) was first proposed by Carl Hueter to reach the hip joint and was later widely used in hip arthroplasty. It has the advantages of less trauma and faster recovery compared to other traditional approaches.

Younger patients are not arthroplasty candidates and there is no consensus on the best fixation implant for FNFs in this population. Many kinds of internal fixators are available for FNFs, such as the cannulated screw (CS), dynamic hip screw (DHS), and compression locking plate. None of these avoid complications or reoperation, so the choice of fixator is controversial. The Femoral Neck System (FNS) is a new type of fixation device designed by a Swiss company for FNFs in young adults [5–7]. However, few relevant studies have been conducted on it. In this study, we evaluated the functional and radiographic outcomes of FNS fixation for irreducible displaced FNFs after open reduction via the DAA approach.

## Patients and methods

### Study population

All patients signed informed consent from January 2020 to September 2021, which was approved by the ethics committee. The inclusion criteria were (1) the imaging diagnosis was femoral neck fracture, (2) the patient age ≤ 55 years old; (3) the follow-up time was longer than 12 months, (4) the fracture of the femoral neck was garden3 ~ 4 type. The exclusion criteria were (1) combined with other site fractures or injuries; (2) combined with severe osteoporosis; (3) a long history of alcohol use or hormone medication; (4) old fracture or pathological fracture; (5) with underlying femoral head ischemia necrosis or hip osteoarthritis. Thus, ultimately, 16 patients with FNFs who received the procedure were included in the study. The preoperative radiological data of the patients were classified according to the Garden classification, which is related to the degree of disrupted blood supply to the femoral head and the incidence of avascular necrosis of the femoral head [8]; 10 patients had Garden III type and 6 patients had Garden IV type. All patients suffered acute injuries and were followed up for at least 12 months after surgery. Among them, 11 were male and 5 were female, with an average age of 34.8 (22–50) years (Table 1).

**Table 1:** Patient demographic and operative data overview

Parameter	Value
Patients (number)	16
Sex Men, number(%)	11 68.75%
Women, number(%)	5 31.25%
Age (years)	34.8(22–50)
BMI (Kg/m2)	26.6(17.5–37.7)
Close fracture, number (%)	16 100%
Open fracture, number (%)	0 0
Mechanism of injury	
Traffic accident, number (%)	10 62.5%
Falling down, number (%)	6 37.5%
Other, number (%)	0 0
Fracture site	
Left, number (%) Right, number (%)	9 56.25% 7 43.75%
Garden grade	
III, number (%) IV, number (%)	10 62.5% 6 37.5%
Diabetes, number (%)	2 12.5%
Duration between injury and surgery (d)	2.1(1–4)

### Surgical technique

**Fig. 1:**
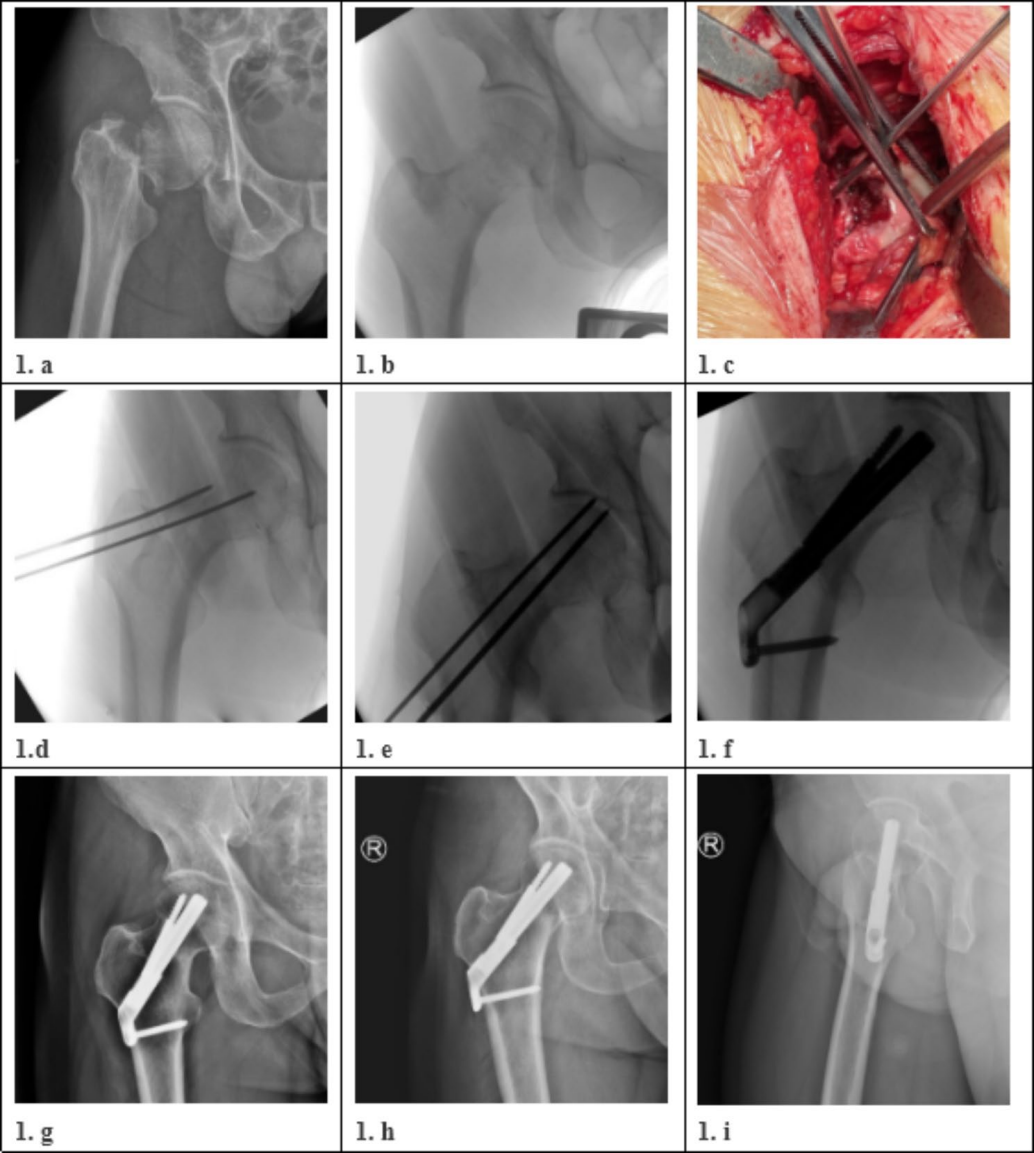
Patient1, Male, fall injury, (1a: Right femoral neck fracture on preoperative X-ray, Garden type IV; 1b: Intraoperative radiography showed that closed reduction could not achieve satisfactory reduction; 1c: Intraoperative incisions image showed that Kirschner wires were inserted into both distal and proximal parts of the fracture to assist reduction; 1d: Intraoperative X-ray showed an anatomic reduction of the fracture; 1e: Intraoperative X-ray showed the insertion of FNS guide needle and an anticockerill needle; 1f: Intraoperative X-ray showed fractures were fixed using FNS and reaching Graden grade I reduction; 1 g: X-ray showed fracture union at 6 months follow-up. 1h1i: At 1 year follow-up, X-ray showed good fracture healing and no ischemic necrosis of femoral head occurred.)

Spinal epidural or general anaesthesia was used with the patient in the supine position. Closed reduction of the FNF was attempted under the traction bed, and those who failed to achieve satisfactory results were included in this study and received an open reduction. Typical cases are shown in Fig. 1.

A 7–10 cm surgical incision was made 2–3 cm outside the anterior superior iliac spine, along the direction of the fibular head. We separated the tensor latissimus muscle and the sartorius muscle space after dissecting the fascia. The ascending branch of the lateral circumflex femoral artery (LCFA) was separated on the lateral side of the rectus femoris muscle, ligated, and severed. A Hoffman hook was used to pull the upper lateral neck of the femur and the large tuberosity of the femur, revealing the joint capsule; a T-shaped incision was made on the capsule of the joint to expose the fracture. The fracture was significantly displaced, so we used a periosteal detacher and other instruments to assist reduction. Kirschner wires were inserted into the femoral head to help control the femoral head (Fig. 1c). Fluoroscopy showed that the FNF was reduced (Fig. 1d). An anti-rotating Kirschner wire was inserted to maintain the reduction and prevent rotation of the femoral head (Fig. 1e). Its position was as close as possible to the upper edge of the femoral neck, and the lateral position was as far away from the centre of the femoral neck as possible.

**Fig. 2:**
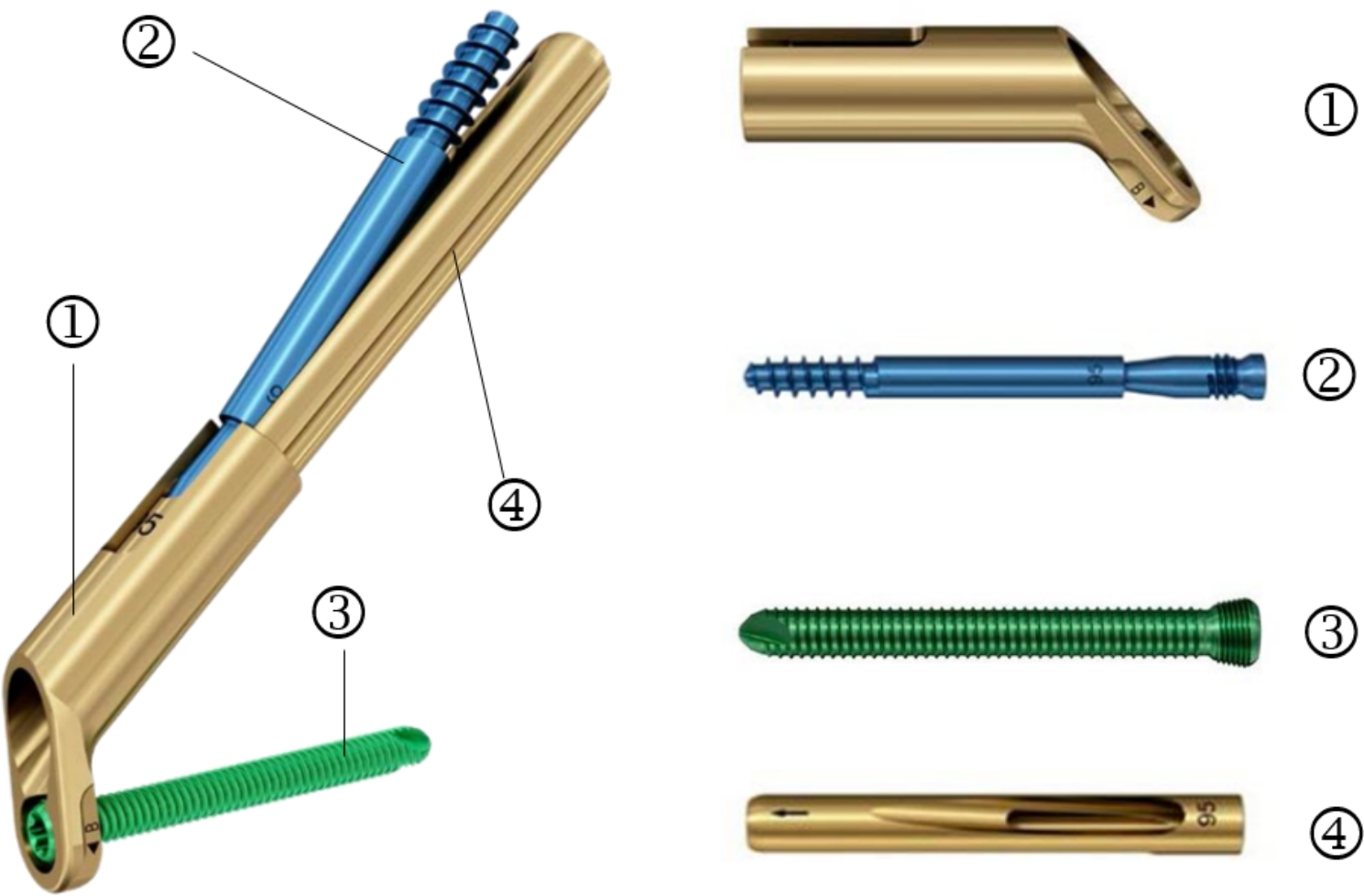
The picture shows the completed and unassembled FNS. (1) pressure plate, (2) anti-rotation screw, (3) distal locking screw, (4) Bolt

**Fig. 3:**
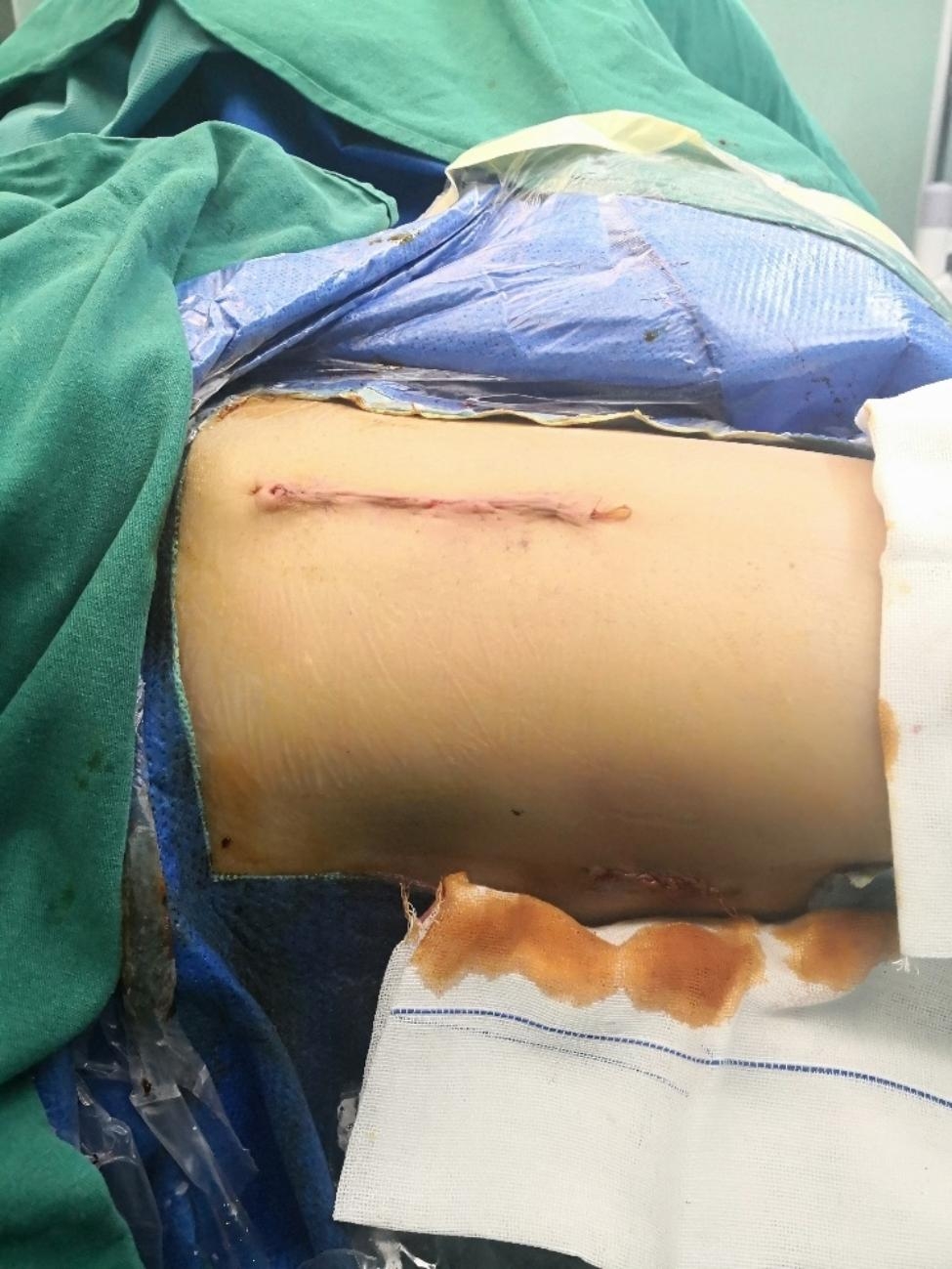
Picture of surgical incision. The upper incision is the incision used for DAA approach, and the lower incision is the incision used for FNS implantation

**Fig. 4:**
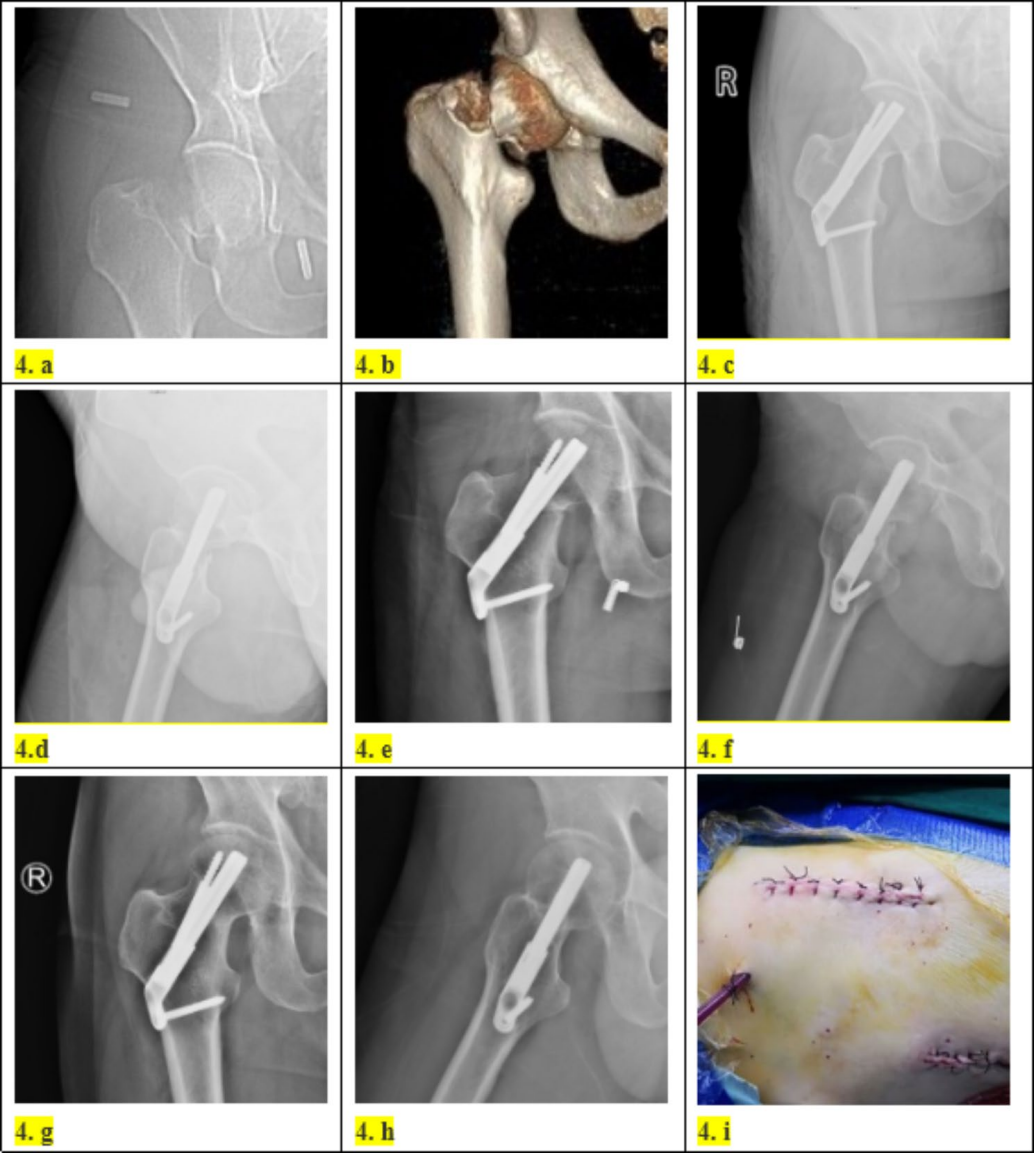
Patient2, Male, Traffic injury, (4a: Right femoral neck fracture on preoperative X-ray, Garden type IV; 4b: Preoperative three-dimensional CT showed a fracture of the right femoral neck; 4c: At 1 month after operation, X-ray films showed good reduction of fracture; 4d: Lateral X-ray films at 1 month after operation showed good reduction of fracture; 4e: At 1 year after operation, X-ray films showed that the fracture healed well; 4f: Lateral X-ray films at 1 year after operation showed that the fracture healed well; 4 g: At 2 years after operation, X-ray films showed that the fractures healed well without avascular necrosis; 4 h: Lateral X-ray films showed good fracture healing without avascular necrosis at 2 years after operation; 4i:Surgical incision diagram.)

Then, a 3–5 cm incision was made at the intersection of the lateral lesser trochanter at the level of the proximal thigh and the lateral longitudinal axis of the femoral shaft. Layer by layer to the lateral cortex of the femur and a guide needle was inserted into the femoral neck with the assistance of a guide device. The tip of the needle was 5 mm from the subchondral area. The angle between the guidewire and the longitudinal axis of the femoral shaft should be 130° as far as possible, and the guidewire was located at the centre of the femoral neck. A femoral neck power rod was gently tapped in after satisfactory positioning. One or two locking screws were drilled into femoral after the lateral femoral plate was attached to the centre of femoral. Finally, an anti-rotation screw of the appropriate length was inserted along the guide. The position of the FNS (DePuy Synthes) (Fig. 2) was examined by fluoroscopy (Fig. 1f), and the anti-Kirschner wire was removed and washed. Drainage was determined according to the intraoperative conditions, and the wound was sutured layer-by-layer and wrapped with a sterile dressing (Fig. 3). A case followed for two years is shown in Fig. 4.

### Evaluation

We collected basic information about the patients such as sex, age, body mass index, injury event, Garden grade, and time from injury to surgery. The Garden classification criteria are as follows: Type I is an incomplete fracture; Type II is a complete fracture without displacement; Type III is a fracture with partial displacement, abduction of the femoral head, mild external rotation, and upward displacement of the femoral neck segment; Type IV is a complete displacement of the femoral neck, being significantly externally rotated and upward.

Clinical outcomes were evaluated using the Harris Hip Score (HHS) [9] at the 12-month follow-up. The HHS score includes assessments of pain, function, deformity, and range of motion, with a score distribution ratio of 44:47:4:5. This tool has been widely used to evaluate hip function. Clinical outcomes were classified as excellent (90–100 points), good (80–89 points), fair (70–79 points), and poor (< 70 points) based on the HHS.

Radiology was performed preoperatively and at the postoperative follow-up, and patients routinely underwent 1-, 2-, 3-, 6-, and 12-month outpatient reviews with plain X-rays. Postoperative reduction was evaluated using the Garden Alignment Index (GAI) on the first day after surgery and 12 months after surgery. The index is determined as follows. The Angle between the inner edge of the femoral shaft and the pressure trabeculae of the medial region of the femoral head in a plain radiograph. A satisfactory Garden index is between 155–180° after reduction.

Avascular necrosis was evaluated by the Steinberg classification; stage 2 and above is avascular necrosis [10]. Postoperative complications, such as infection, deep vein thrombosis (DVT), and postoperative internal fixation failure were noted. Non-union was judged according to the criteria described by Dha*r et al.* [11]. The degree of shortening of the femoral neck was categorised as none/mild, < 5 mm; moderate, 5–10 mm, and severe, > 10 mm [5].

### Statistical analysis

Data are presented as mean ± standard deviation. The paired samples *t*-test was used to evaluate differences in the preoperative and postoperative outcomes for continuous variables. A P-value < 0.05 was considered significant. Statistical analysis was performed with SPSS version 25.0 software (SPSS Inc., Chicago IL, USA).

## Results

All patients achieved anatomic reduction. All patients were followed up for at least 12 months, with a mean follow-up of 21.1 (12–30) months. Based on the radiological results, all fractures healed, with an average healing time of 4.25 (3–6) months. According to X-rays of hips taken on the first day after surgery and 12 months after surgery, all 16 patients achieved Garden I and II reduction, and no significant difference was observed between the time points. The postoperative HHS score significantly improved after the surgery. Excellent outcomes were achieved in 13 cases. No or mild femoral neck shortness occurred in 12 (75%) of 16 patients, moderate shortening occurred in three (18.75%) patients, and severe shortening occurred in one (6.25%) patient. One patient developed lower limb DVT after surgery and received an inferior vena cava filter. None of the patients developed avascular necrosis or infection at the incision site, experienced non-union of a fracture, or had any internal fixations fail (Table 2).

**Table 2:** The outcomes and complications of preoperatively and postoperatively (mean and standard deviation (SD))

Parameter	Preoperation	Postoperatiom		*P* value
Clinical evaluation				
HHS score	17.19 ± 4.8	92.19 ± 3.4		0.000
Excellent	13 patients (81.25%)			
Good	3 patients (18.75%)			
Fair	0 (0)			
Poor	0 (0)			
Complications				
DVT		1 patient (6.25%)		
Avascular necrosis Incision infection		0 (0) 0 (0)		
nonunion fracture		0 (0)		
Femoral neck shortening				
<5 mm	12 (75%)			
5-10 mm	3 (18.75%)			
>10 mm	1 (6.25%)			
Fluoroscopy times	6.31 ± 1.7			
**Parameter**	**Preoperation**	**Postoperatiom(1d)**	**Postoperatiom(12month)**	***P*** **value**
Radio evaluation				
Garden Index (Anteroposterior)		166.13 ± 5.61	164.94 ± 4.49	0.462
Garden Index (Lateral)		171.06 ± 4.46	169.38 ± 3.98	0.247

## Discussion

FNFs are common in the elderly, which may be related to osteoporosis or other ailments [12]. In young people, such fractures usually occur due to high-energy trauma. The postoperative healing and necrosis rates of the femoral head are important indices to evaluate the curative effect of FNF surgery. The degree of displacement of the fracture and the quality of the fracture reduction are closely related to postoperative healing. According to a previous meta-analysis, varus deformities of the femoral head after fracture reduction are associated with significantly higher postoperative non-union rates [13]. Beris et al. reported that fracture reduction treatment is the primary factor affecting femoral head necrosis [14]. Among the various treatment modalities, closed reduction and immobilisation appear to be the less destructive; however, an anatomic reduction can be difficult to achieve using the closed method and may lead to osteonecrosis of the femoral head after multiple attempts. Thus, open reduction and anatomic reduction may be ideal. In this study, we changed to open reduction immediately if a closed reduction was difficult to achieve.

The surgical field is fully exposed during open reduction, the internal fixation is placed under direct vision, and the number of intraoperative fluoroscopies decreases. In our study, mean fluoroscopy time was 6.31 ± 1.7. He et al. [15] and Zhou et al. [5] also reported that using FNS to fix FNFs reduced the number of intraoperative fluoroscopies, which reduces radiation exposure.

Fixation of the femoral neck is usually done using Smith-Petersen approach (S-P approach) and the Watson-Jones approach (W-J approach). However, the DAA approach has also been reported be useful for hip joint arthroplasty [16]. We noted two benefits of using the DAA approach for treating FNFs. First, it effectively reduces damage to the surrounding soft tissues through the gaps between the tensor fascia lata and the sartorius muscle and between the rectus femoris and the gluteus medius. Second, it entails entering through the anterior area of the hip joint, which avoids hindering the blood supply to the femoral head, thereby prevent necrosis of the femoral head.

Dewar et al. reported that 82% of the blood supply to the femoral head comes from the medial femoral circumflex artery [17], making it the main blood supply source to the head and neck of the femur; this vessel is not damaged when performing the DAA approach. Intraoperative ligation of the lateral femoral circumflex artery (LCFA) does not affect the blood supply to the head and neck [18]. About 10–20% and 10–30% of patients have complications of non-union and avascular necrosis, respectively [19, 20]. In this study, none of the patients had developed cystic degeneration or peripheral sclerosis of the femoral head at the last postoperative follow-up, and none developed incision infection or delayed healing after surgery. This result may be related to better protection of the blood supply to the femoral head when using the DAA approach.

The most common internal fixation methods for an FNF are the CS and DHS. CS fixation provides torsional stability and less damage to the blood supply to the femoral head, but using multiple screws results in higher failure rates when the fracture is displaced due to vertical shear stress; therefore, an internal fixation system with a fixed angle device, such as the DHS, is preferred for this type of fracture. The FNS system combines the fixed angle stability of the DHS with the minimally invasive advantages of CS. Stoffel et al. showed that the FNS is an effective alternative for treating unstable FNFs, with minimally invasive implantation and good biomechanical stability compared to DHS systems and superior to CS [21]. A biomechanical study also suggested that the FNS is more stable than the use of three screws [22], karl et al. also indicated in biomechanical tests that FNS had better overall structural stability than internal fixation with three hollow screws [23]. Xu et al. used FNS to treat 16 patients with femoral neck fracture, and the follow-up results were satisfactory without related complications [24]. In this clinical study, no screw withdrawal or loosening occurred in any case. The fixation effect of the FNS system was clear and the fixation outcome was good.

A shortening deformity of an FNF after internal fixation is a common phenomenon, and may even be a factor associated with joint arthroplasty [25]. In our study, 75% of patients had no/mild shortening, 18.75% had moderate shortening, and 6.25% had severe shortening at the last follow-up. As previously reported, there is a high incidence of shortening after CS fixation of an FNF. In one study, moderate and severe shortening occurred in 22.8% and 12.9% of young adults who underwent screw fixation of FNFs [26]. Whether femoral neck shortening affects hip joint function remains controversial. Some scholars believe that any deformity arising from the procedure would harm hip joint function [27, 28]. The HHS is an indicator of hip pain, function, range of motion, and other activities. In this study, the HHS score was significantly higher after surgery, indicating that the patients achieved satisfactory surgical results through FNS fixation under the DAA approach.

All patients achieved Garden Grade I and II reduction. Only one patient developed DVT of a lower extremity after surgery, and no other complications, such as a non-union fracture or postoperative incision infection, were observed. In the study on the treatment of femoral neck fractures in young adults, zhou et al. found that the use of FNS had significant advantages in early clinical efficacy and complication rate compared with traditional screw fixation, and significantly reduces the operation time [6]. The use of FNS can effectively reduce soft tissue exposure, and usually only requires a 4-5 cm lateral incision to complete the insertion. In addition, it does not damage the gluteus medius muscle. Less trauma and fewer complications indicate that FNS treatment is a simple and effective treatment.

In conclusion, the DAA approach combined with FNS achieved satisfactory results for FNFs that could not be fixed under closed reduction. However, our study had some limitations. It was a retrospective study with a small sample size and a short follow-up time. Also, we did not follow up on long-term functional outcomes and whether revision surgery was performed, Finally, there was insufficient statistical power because there was no control group.

## Data Availability

Data and materials were available from the corresponding author.

## References

[1] Bhandari M, Swiontkowski M (2017). Management of Acute Hip fracture. N Engl J Med.

[2] Bensen AS, Jakobsen T, Krarup N (2014). Dual mobility cup reduces dislocation and re-operation when used to treat displaced femoral neck fractures. Int Orthop.

[3] Elgeidi A, El Negery A, Abdellatif MS, El Moghazy N (2017). Dynamic hip screw and fibular strut graft for fixation of fresh femoral neck fracture with posterior comminution. Arch Orthop Trauma Surg.

[4] Kang JS, Moon KH, Shin JS, Shin EH, Ahn CH, Choi GH (2016). Clinical results of internal fixation of Subcapital femoral Neck Fractures. Clin Orthop Surg.

[5] Tang Y, Zhang Z, Wang L, Xiong W, Fang Q, Wang G (2021). Femoral neck system versus inverted cannulated cancellous screw for the treatment of femoral neck fractures in adults: a preliminary comparative study. J Orthop Surg Res.

[6] Zhou XQ, Li ZQ, Xu RJ, She YS, Zhang XX, Chen GX, Yu X (2021). Comparison of early clinical results for femoral Neck System and Cannulated Screws in the treatment of unstable femoral Neck Fractures. Orthop Surg.

[7] Hu H, Cheng J, Feng M, Gao Z, Wu J, Lu S (2021). Clinical outcome of femoral neck system versus cannulated compression screws for fixation of femoral neck fracture in younger patients. J Orthop Surg Res.

[8] Konishiike T, Makihata E, Tago H, Sato T, Inoue H (1999). Acute fracture of the neck of the femur. An assessment of perfusion of the head by dynamic MRI. J Bone Joint Surg Br.

[9] Harris WH (1969). Traumatic arthritis of the hip after dislocation and acetabular fractures: treatment by mold arthroplasty. An end-result study using a new method of result evaluation. J Bone Joint Surg Am.

[10] Steinberg ME, Hayken GD, Steinberg DR (1995). A quantitative system for staging avascular necrosis. J Bone Joint Surg Br.

[11] Dhar SA, Gani NU, Butt MF, Farooq M, Mir MR (2008). Delayed union of an operated fracture of the femoral neck. J Orthop Traumatol.

[12] Parker M, Johansen A (2006). Hip fracture. BMJ.

[13] Dai Z, Li Y, Jiang D (2011). Meta-analysis comparing arthroplasty with internal fixation for displaced femoral neck fracture in the elderly. J Surg Res.

[14] Beris AE, Payatakes AH, Kostopoulos VK, Korompilias AV, Mavrodontidis AN, Vekris MD, Kontogeorgakos VA, Soucacos PN (2004). Non-union of femoral neck fractures with osteonecrosis of the femoral head: treatment with combined free vascularized fibular grafting and subtrochanteric valgus osteotomy. Orthop Clin North Am.

[15] He C, Lu Y, Wang Q, Ren C, Li M, Yang M, Xu Y, Li Z, Zhang K, Ma T (2021). Comparison of the clinical efficacy of a femoral neck system versus cannulated screws in the treatment of femoral neck fracture in young adults. BMC Musculoskelet Disord.

[16] Rodriguez JA, Deshmukh AJ, Rathod PA, Greiz ML, Deshmane PP, Hepinstall MS, Ranawat AS (2014). Does the direct anterior approach in THA offer faster rehabilitation and comparable safety to the posterior approach?. Clin Orthop Relat Res.

[17] Dewar DC et al. The relative contribution of the medial and lateral femoral circumflex arteries to the vascularity of the head and neck of the femur: a quantitative MRI-based assessment. Bone Joint J, 2016. 98–b(12): p. 1582–1588. 10.1302/0301-620X.98B12.BJJ-2016-0251.R1 27909118

[18] Kalhor M, Beck M, Huff TW, Ganz R (2009). Capsular and pericapsular contributions to acetabular and femoral head perfusion. J Bone Joint Surg Am.

[19] Angelini M, McKee MD, Waddell JP, Haidukewych G, Schemitsch EH (2009). Salvage of failed hip fracture fixation. J Orthop Trauma.

[20] Haidukewych GJ, Rothwell WS, Jacofsky DJ, Torchia ME, Berry DJ (2004). Operative treatment of femoral neck fractures in patients between the ages of fifteen and fifty years. J Bone Joint Surg Am.

[21] Stoffel K, Zderic I, Gras F, Sommer C, Eberli U, Mueller D, Oswald M, Gueorguiev B (2017). Biomechanical evaluation of the femoral Neck System in Unstable Pauwels III femoral Neck Fractures: a comparison with the dynamic hip screw and cannulated screws. J Orthop Trauma.

[22] Fan Z (2021). How to choose the suitable FNS specification in young patients with femoral neck fracture: a finite element analysis. Injury.

[23] Stoffel K, Zderic I, Gras F (2017). Biomechanical evaluation of the femoral neck system in unstable Pauwels III femoral neck fractures: a comparison with the dynamic hip screw and cannulated screws. J Orthop Trauma.

[24] Xu XZ, Chang J, Yu SS (2020). Fixation with femoral neck system for femoral neck fractures: short-term therapeutic outcomes. Chin J Orthop Trauma.

[25] Pauyo T, Drager J, Albers A, Harvey EJ (2014). Management of femoral neck fractures in the young patient: a critical analysis review. World J Orthop.

[26] Slobogean GP, Stockton DJ, Zeng BF, Wang D, Ma B, Pollak AN (2017). Femoral neck shortening in adult patients under the age of 55 years is associated with worse functional outcomes: analysis of the prospective multi-center study of hip fracture outcomes in China (SHOC). Injury.

[27] Linde F, Andersen E, Hvass I, Madsen F, Pallesen R (1986). Avascular femoral head necrosis following fracture fixation. Injury.

[28] Zielinski SM, Keijsers NL, Praet SF, Heetveld MJ, Bhandari M, Wilssens JP, Patka P, Van Lieshout EM (2013). Femoral neck shortening after internal fixation of a femoral neck fracture. Orthopedics.

